# Comparative Analysis of KnockOut*™* Serum with Fetal Bovine Serum for the In Vitro Long-Term Culture of Human Limbal Epithelial Cells

**DOI:** 10.1155/2016/7304812

**Published:** 2016-06-30

**Authors:** Shaokun Zhang, Zaoxia Liu, Guanfang Su, Hong Wu

**Affiliations:** ^1^Department of Orthopedics, The 1st Hospital of Jilin University, Changchun 130021, China; ^2^Department of Ophthalmology, The 2nd Hospital of Jilin University, Changchun 130041, China

## Abstract

The limbal epithelial cells can be maintained on 3T3 feeder layer with fetal bovine serum supplemented culture medium, and these cells have been used to successfully treat limbal stem cell deficiency. However, fetal bovine serum contains unknown components and displays quantitative and qualitative lot-to-lot variations. To improve the culture condition, the defined KnockOut serum replacement was investigated to replace fetal bovine serum for culturing human limbal epithelial cell. Human primary limbal epithelial cells were cultured in KnockOut serum and fetal bovine serum supplemented medium, respectively. The cell growth rate, gene expression, and maintenance of limbal epithelial stem cells were studied and compared between these two groups. Human primary limbal epithelial cells were isolated and successfully serially cultivated in this novel KnockOut serum supplemented medium; the cell proliferation and stem cell maintenance were similar to those of cells grown in fetal bovine serum supplemented medium. These data suggests that this KnockOut serum supplemented medium is an efficient replacement to traditional fetal bovine serum supplemented medium for limbal epithelial cell culture, and this medium has great potential for long term maintenance of limbal epithelial cells, limbal epithelial stem cells transplantation, and tissue regeneration.

## 1. Introduction

Corneal epithelial stem cells are located in the basal layer of the limbus, a corrugated and pigmented structure called the palisades of Vogt [1–4]. These stem cells sustain the continuous renewal of the corneal epithelium over a lifetime and replace injured or lost corneal epithelial cells [5, 6]. Limbal stem cell deficiency (LSCD) and associated ocular surface diseases can be treated successfully using cultured limbal epithelial autograft [7, 8]. The success of these surgical treatments depends on efficient expansion of limbal epithelial stem cells which involved 3T3 feeder layer and fetal bovine serum (FBS) in most cases. The 3T3 feeder layer culture system was set up by Rheinwald and Green [9] and has been successfully used to expand epithelial cells from human skin, hair follicle, limbus, conjunctiva, and oral mucosa tissue [10–16]. However, FBS is not well-defined, and it always displays quantitative and qualitative lot-to-lot variations [17]. FBS also contains potentially harmful xenogeneic components, which may stimulate immunological reactions and transmit animal diseases and pathogens [18]. With all these concerns, there is an increasing need to develop well-defined culture medium to replace the traditional FBS supplemented medium.

Currently there are certain serum-free alternative media for the growth of epithelial cells, such as defined Keratinocyte Serum-Free Medium (KSFM*™*, Invitrogen, USA), keratinocyte growth medium (KGM*™*, Clontech, USA), Epilife*™* (Invitrogen, USA), and Progenitor Cell Targeted (PCT) media (CellnTEC*™*, Switzerland). These products have been shown to support the expansion of corneal epithelial cells. However, they still need supplements of undefined products, such as bovine pituitary extract (BPE) or human serum albumin (HAS). And most of these media require high cell seeding density, which may not be practical for expansion of human corneal epithelial cells. Moreover, corneal epithelial stem cells, which are detected as holoclones in 3T3 culture system, could not be maintained in this serum-free culture medium for long term [12, 13]. To date, there is no defined serum-free medium which could support expansion of corneal epithelial stem cells for long term.

KnockOut serum replacement (SR) is a defined serum-free formulation, which was designed to directly replace FBS for maintenance of embryonic stem cells (ESCs) and induced pluripotent stem cells (iPSCs). It has been demonstrated that KnockOut SR provides consistent growth conditions for ES cell and iPSC culture, and ES cells grown in KnockOut SR supplemented medium are substantially less differentiated than those grown in FBS supplemented medium, and germline transmission is not compromised in the least. Given the similarities in the culture methods of epithelial cells and ES cells, and in light of the fact of KnockOut SR replacing FBS in ES cell culture, here it is hypothesized that the KnockOut SR could be used to replace FBS in limbal epithelial cell culture.

## 2. Materials and Reagents

Cell culture dishes, flasks, centrifuge tubes, and serological pipettes were purchased from Becton Dickinson (Franklin Lakes, NJ; http://www.bd.com/). Dulbecco's Modified Eagle's Medium (DMEM), Ham's F-12, HEPES, penicillin and streptomycin, L-glutamine, 0.05% trypsin-0.02% EDTA solution, Superscript III kit, and RNeasy*™* kit were acquired from Invitrogen-GIBCO BRL (Grand Island, NY; http://www.invitrogen.com/). Fetal bovine serum (FBS) was purchased from Hyclone (Logan, UT; http://www.hyclone.com/). Mouse NIH 3T3 fibroblasts (ATCC CCL 92) were obtained from American Type Culture Collection (Rockville, MD; http://www.atcc.org/). Dispase II was from Roche. Monoclonal antibody (mAb) against ABCG2 (clone BXP-21) and connexin 43 were from Millipore; p63 (clone 4A4), K5, and K19 came from Santa Cruz; K3 mAb (clone AE5) was from ICN Pharmaceuticals (Costa Mesa, CA; http://www.mpbio.com/). Alexa Fluor 568-conjugated goat anti-mouse secondary antibody was from Invitrogen-GIBCO BRL (Grand Island, NY; http://www.invitrogen.com/). GeneAmp RNA-PCR and Taqman Universal PCR Master Mix AmpErase UNG kits were from Applied Biosystems (Foster City, CA; http://www.appliedbiosystems.com/). Mitomycin C, bovine insulin, human transferrin, hydrocortisone, human epidermal growth factor (EGF), cholera toxin, and other reagents were from Sigma-Aldrich (St. Louis; http://www.sigmaaldrich.com/).

### 2.1. Human Limbal Epithelial Cell Isolation and Cultivation

Cornea-limbal rings were harvested from five healthy donors just after corneal transplantation, informed consent was sought, and the sample harvesting protocol was approved by the Institutional Review Board (IRB) of Jilin University. Fresh cornea-limbal rings were treated with 0.25% Dispase II at 4°C overnight, and epithelial layer was scrubbed from the underlying stroma tissue and treated with 0.05% trypsin-0.02% EDTA at 37°C for 15 minutes. Trypsin activity was neutralized by 10% FBS and dissociated limbal epithelial cells were collected and centrifuged at 1,500 rpm for 5 minutes. Epithelial cell viability was determined by trypan blue excluding staining and cell number was counted using hemocytometer.

Mouse 3T3 fibroblasts were maintained in Dulbecco's Modified Eagle's Medium (DMEM, high glucose) supplemented with 10% FBS, L-glutamine (2 mM), and penicillin-streptomycin (50 IU/mL) and cultured with 5% CO_2_ and humidified atmosphere. 3T3 cells were subcultured every 6 days when reaching 80–90% confluence. 3T3 cells were serially maintained, and only cells before passage 20 were used for preparation of feeder layer. To prepare feeder layer, confluent 3T3 cells were treated with mitomycin C (10 *μ*g/mL) for 2 hours at 37°C, washed with PBS twice, and treated with 0.05% trypsin for 5 minutes at 37°C. 3T3 fibroblasts were then collected and plated at a density of 30,000 cells/cm^2^ one day before seeding epithelial cells.

Human limbal epithelial cells were cultivated on 3T3 feeder layer using either FAD medium or serum replacement (SR) medium. The FAD medium is a mixture of DMEM and Ham's F-12 medium (1 : 1) containing 10% fetal bovine serum, L-glutamine (2 mM) and penicillin-streptomycin (50 IU/mL), epidermal growth factor (10 ng/mL), insulin (5 *μ*g/mL), adenine (0.18 mM), hydrocortisone (0.4 *μ*g/mL), cholera toxin (0.1 nM), and triiodothyronine (2 nM). The SR medium is a mixture of DMEM and Ham's F-12 medium (1 : 1) containing 10% KnockOut SR serum replacement, L-glutamine (2 mM) and penicillin-streptomycin (50 IU/mL), epidermal growth factor (10 ng/mL), insulin (5 *μ*g/mL), adenine (0.18 mM), hydrocortisone (0.4 *μ*g/mL), cholera toxin (0.1 nM), triiodothyronine (2 nM), transferrin (5 *μ*g/mL), and selenium (5 ng/mL). Limbal epithelial cells were seeded onto 3T3 feeder layer at a density of 6,000 cells/cm^2^ and cultured with 5% CO_2_ and humidified atmosphere. The FAD medium was changed every 3 days while the SR medium was changed every 2 days.

### 2.2. Colony Forming Efficiency Assay (CFE)

For CFE assay, 200 human limbal epithelial cells from FAD culture or SR culture were plated onto 100 mm petri dish containing mitomycin C-treated 3T3 feeder layer and cultivated as described above. After 12-day culture, the dishes were fixed with 10% formalin for 30 minutes at room temperature and stained with 1% Rhodamine B for another 30 minutes. After washing with distilled water, the colony numbers were counted and analyzed. The colony forming efficiency was expressed as the number of colonies formed divided by 200.

### 2.3. Cell Population Doubling Assay

Limbal epithelial cells were maintained on mitomycin C-treated 3T3 feeder layer using FAD medium or SR medium. Epithelial cells were trypsinized when reaching 80–90% confluence and passaged at a density of 6,000 cells/cm^2^. Cultures were serially maintained for 10 passages. At each passage, limbal epithelial cells were harvested and cell numbers were counted. The number of population doublings (PD) for each passage was calculated according to the following formula: PD = (log⁡*N*/*N*_0_)/log⁡2, where *N* represents the total number of cells obtained at each passage and *N*_0_ represents the number of plating cells at the beginning.

### 2.4. RNA Extraction and Quantitative Real-Time Polymerase Chain Reaction

To evaluate the gene expression level of limbal epithelial cells, PCR and real-time PCR were performed. Total RNA was extracted from the corneal epithelial cells using the RNeasy kit following the manufacturer's instructions. The RNA was quantified by its absorption at 260 nm and stored at −80°C. To synthesize cDNAs, 1 *μ*g of total RNA was used with Superscript III Reverse Transcription kit. PCR was performed using Platinum PCR master mix (Life Technology); and real-time PCR was performed using the SYBR Green PCR Master Mix (Roche) with the primers described before [19, 20]. Real-time PCR reactions were performed in triplicate with an initial activation step at 95°C for 3 minutes, followed by 45 cycles: 95°C for 30 seconds, 60°C for 30 seconds, and 72°C for 40 seconds. Relative gene expression was calculated by normalizing the difference in cycle threshold value (delta Ct), values of the genes to the delta Ct value of glyceraldehydes-3-phosphate dehydrogenase (GAPDH).

### 2.5. Immunofluorescent Staining

Human limbal epithelial cells were seeded in culture slides with 3T3 feeder layer in FAD medium or SR medium. Three days after seeding, cultures were fixed with 4% paraformaldehyde or cold acetone at 4°C for 10 minutes. After washing with PBS twice, cells were blocked with 5% goat serum in PBS for 30 minutes. The mouse primary antibodies against p63 (1 : 200, 4A4, Santa Cruz), ABCG2 (1 : 30, BXP-21, Millipore), K3 (1 : 400, Millipore), and connexin 43 (1 : 1000, Millipore) were applied and incubated overnight at 4°C. Alexa Fluor 568-conjugated secondary antibody was applied for 1 hour after washing with PBS twice. The cell nuclei were then counterstained with Hoechst 33342 (1 *μ*g/mL in PBS) for 20 minutes. After washing with PBS for 3 times, the cell culture was mounted with mounting medium (Vector Laboratories, Burlingame, CA) and examined under a fluorescent microscope (BX50; Olympus, Tokyo, Japan).

### 2.6. Western Blot Analysis

Cultured limbal epithelial cells were lysed with RIPA buffer (10 mM Tris pH 7.5, 150 mM NaCl, 1% sodium deoxycholate, 1% Triton X-100, 1 mM EDTA, and protease inhibitor cocktail; Roche Diagnostics) on ice for 30 minutes. The protein concentration was quantified using bicinchoninic acid (BCA) protein assay (Pierce, Rockford, IL). Total cell lysates (40 *μ*g) were electrophoresed in 12% gradient SDS-PAGE gel, transferred to nitrocellulose membrane (Bio-Rad), blocked with 5% skimmed milk in Tris-buffered saline (TBS) for 1 hour, and probed with mouse antibodies against proliferating cell nucleus antigen (PCNA, Santa Cruz, CA), ABCG2 (BXP-21, Millipore), or p63 (4A4, Santa Cruz, CA) overnight at 4°C. The membranes were then washed three times with TBS, incubated with goat anti-mouse IgG (1 : 5000, HRP-conjugated, Santa Cruz, CA) or rabbit anti-goat IgG (1 : 5000, HRP-conjugated, Santa Cruz, CA) for 1 hour at room temperature, and developed with enhanced chemiluminescent substrates (Pierce Biotechnology, Rockford, IL). The expression levels of ABCG2, PCNA, and p63 were measured using semiquantitative intensity measurement and normalized to GAPDH level which served as internal control.

### 2.7. Statistics

Summary of data of CFE and relative fold of real-time PCR was reported as means ± SD and compared using Student's unpaired *t*-test with Microsoft Excel (2003/XP version). Test results were reported as two-tailed *p* values, where *p* < 0.05 was considered statistically significant.

## 3. Results

### 3.1. The Phenotype of Corneal Epithelial Stem Cells in SR Medium

**Figure 1:**
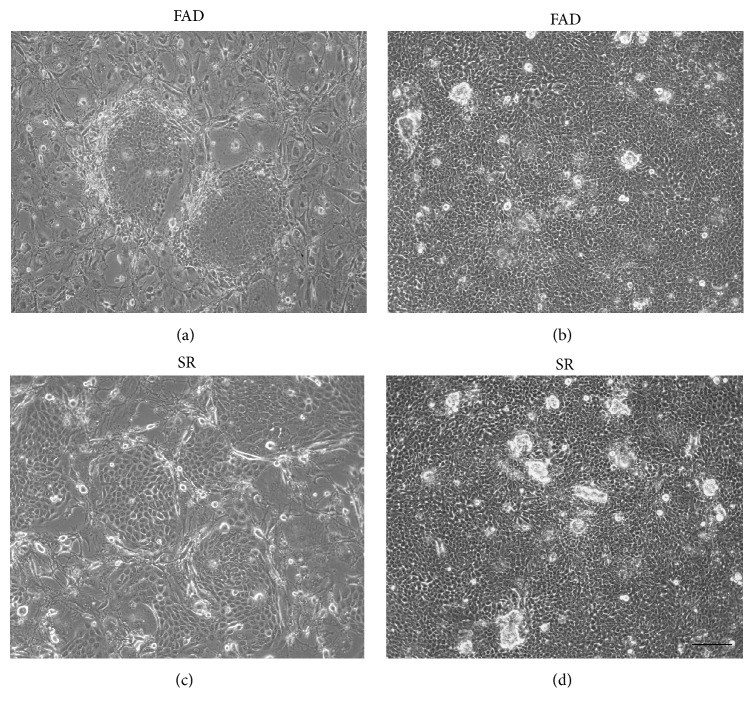
The culture of human limbal epithelial cells in SR medium. (a) The human corneal epithelial cells formed tightly compacted colonies in FAD medium after seeding cells for 3 days. (b) After 7 days, epithelial cells reached confluence in FAD medium with uniform small size. (c) Human corneal epithelial cells formed colonies within SR medium, which were similar to those formed within FAD medium, but these colonies were less tightly compacted than those in FAD. (d) After 7 days, epithelial cells reached confluence in SR medium with uniform small size which was similar to those in FAD culture Original magnification: (a) ×10; (b) ×10 (scale bars: 100 *μ*m).

**Figure 2:**
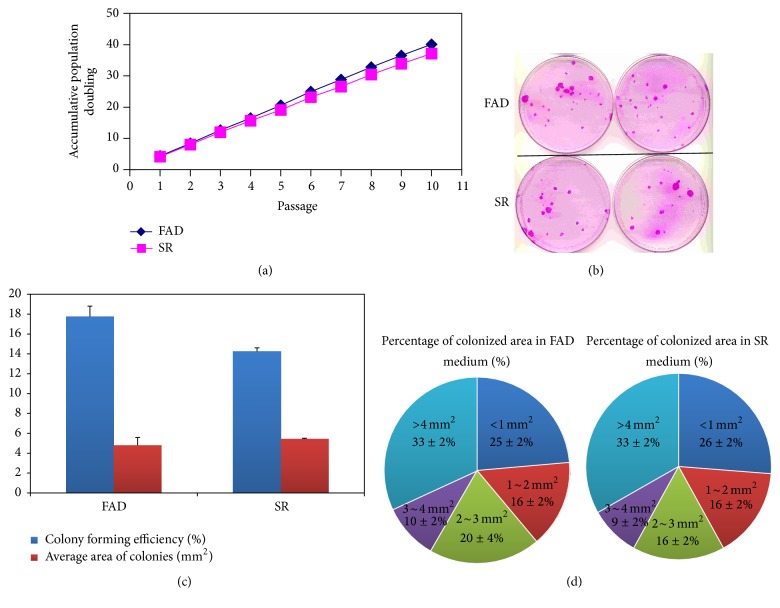
Comparison of cell population doubling (PD) and colony forming efficiency (CFE) of human limbal epithelial cells cultured in SR and FAD media. (a) Human limbal epithelial cells were serially subcultured in SR medium and FAD medium for 10 passages (*n* = 5); cell population doublings (PD) number and accumulated PD were calculated and plotted. PD was calculated according to the following formula: PD = (log⁡*N*/*N*_0_)/log⁡2, where *N* represents the total number of cells obtained at each passage and *N*_0_ represents the number of plating cells at the beginning of each passage. (b) To analyze the CFE, limbal epithelial cells were seeded at a density of 200 cells per 100 mm petri dish in SR and FAD media and allowed to grow for 12 days. (c) CFE values for epithelial cells in SR and FAD media were 17% ± 2.5% and 15% ± 3.1%, respectively. No differences of CFE were found between SR medium and FAD medium group (*p* > 0.05, *n* = 5). (d) Percentage of colonized area in FAD was close to that of SR medium. The limbal epithelial cells maintained in SR medium exhibited similar CFE and colony size compared to cells maintained in FAD medium.

A total of 5 cornea-limbal ring tissues were harvest from donors in the age range of 32–65 years. These tissues were preserved and processed within 24 hours after harvest. Human primary corneal epithelial cell culture was successfully set up in FAD medium (Figures 1(a) and 1(b)) and SR medium (Figures 1(c) and 1(d)) as well. The corneal epithelial cells maintained in SR medium + 3T3 feeder layer displayed a morphology with small size and high nuclei/cytoplasm ratio, which was typical undifferentiated epithelial cells morphology, and the large differentiating flat squamous-like cells were rarely observed (Figure 1(c)). The epithelial cells maintained in SR medium began to form colonies 3 days after seeding (Figure 1(c)). The sizes of these colonies were similar to those formed within FAD medium + 3T3 feeder layer (Figure 1(a)), but these colonies were less tightly compacted than those in FAD + 3T3 feeder layer. Within 7 days, epithelial cells reached confluence in SR medium with uniform small size (Figure 1(d)), which was also observed in FAD culture (Figure 1(b)). In this study, human corneal epithelial cells could be derived and maintained in SR medium + 3T3 feeder layer for all five donors. For long-term cultivation, human corneal epithelial cells were serially subcultured in SR medium + 3T3 feeder layer for more than 10 passages (*n* = 5). During each passage, cells were trypsinized and collected when cells reached 80–90% confluence; cell numbers were calculated for population doubling assay. The result shows that epithelial cell yield from SR medium is close to that of cells cultured in FAD medium, as shown in Figure 2(a), and these data are similar to previous reports [4, 10–12]. In summary, the data presented here shows that SR culture medium + mitomycin C-treated 3T3 feeder layer supports human corneal epithelial cells clonal growth and proliferation.

### 3.2. CFE Assay

Next we examined and compared the CFE of corneal epithelial cells cultured in FAD and SR media. To analyze the CFE, corneal epithelial cells cultured in SR and FAD media were harvested and seeded at a density of 200 cells per 100 mm petri dish, which was preseeded with mitomycin C-treated 3T3 feeder layer, and this culture was allowed to grow for 12 days. The corneal epithelial cells started to form colonies 5–7 days after culture initiation. As shown in Figure 2(b), after culturing for 12 days, the corneal epithelial cells maintained in SR medium exhibited similar CFE and colony size compared to cells maintained in FAD medium. CFE value for epithelial cells in SR and FAD media was 15% ± 3.1% and 17% ± 2.5%, respectively (Figure 2(c)). Statistical analysis demonstrated that there was no significant differences in colony forming efficiency (*p* > 0.05) and the average area of the colonies (*p* > 0.05) between SR and FAD media (Figure 2(c)). We quantified the percentage of the colony types based upon the area of the colonies. The results showed that the percentage of abortive colonies (colony area <1 mm^2^) formed in SR medium was similar to that within FAD medium (*p* > 0.05). Similarly, the percentage of large colonies (colony area >4 mm^2^) formed in SR medium was close to that in FAD medium (*p* > 0.05) (Figure 2(d)). These results indicate that corneal epithelial cells cultured in SR medium possess a similar higher degree of proliferative potential when compared to cells grown in FAD medium.

### 3.3. PCR and Real-Time PCR Analysis

To study the gene expression, PCR and real-time PCR were performed on epithelial cells cultured in both FAD and SR media. Housekeeping gene GAPDH was used as an internal control. PCR data shows that the cell cultured in both FAD medium and SR medium show high level transcript expression of proliferative marker PCNA. The cells in both media show positive expression of cytokeratin 3 and cytokeratin 12, which is consistent with their limbus origin. The positive expression of basal layer epithelial cell marker, cytokeratin 15, was also observed in both cultures. ABCG2 and ΔNp63*α* expression was detected in both cultures, although ABCG2 expression decreased slightly after primary culture in SR medium. The low level of connexin 43 expression was observed in both cultures and implied low level of epithelial cell differentiation in both media. For real-time PCR, quantitative analysis of the mRNA level showed that there was no significant expression difference (*p* > 0.05) of p63 and ABCG2 between epithelial cells grown in FAD and SR media (Figure 4(a)). Real-time analysis of the corneal epithelial differentiation marker cytokeratin 3 and cytokeratin 12 was also performed. Both of these two markers were barely detectable in both FAD and SR cell cultures, and, surprisingly, there is no significant difference (*p* > 0.05) in expression level between these two cultures.

### 3.4. Immunofluorescent Staining

To confirm that the epithelial cells grown in SR medium maintained the stemness and undifferentiated state, immunofluorescent staining of several proliferation, differentiation, and putative epithelial stem cell markers was performed. As shown in Figure 4, in SR medium, most of epithelial cells displayed a small and uniform morphology, and most cells were strongly stained with p63, the putative epithelial stem cell marker. ABCG2 positive stained cells were also observed in a patchy distribution within the limbal epithelial cell colonies. The cells maintained in SR medium were weakly stained for connexin 43 and cytokeratin 3. The low expression of these two differentiation markers suggests low level of epithelial cells differentiation in the cell culture. The similar staining style was also observed in cells maintained with FAD medium (Figure 4(b)). These data implies that human limbal epithelial cells cultured in SR medium maintained high level expression of putative epithelial stem cell markers, while expressing low level of differentiation markers.

### 3.5. Western Blot

**Figure 3:**
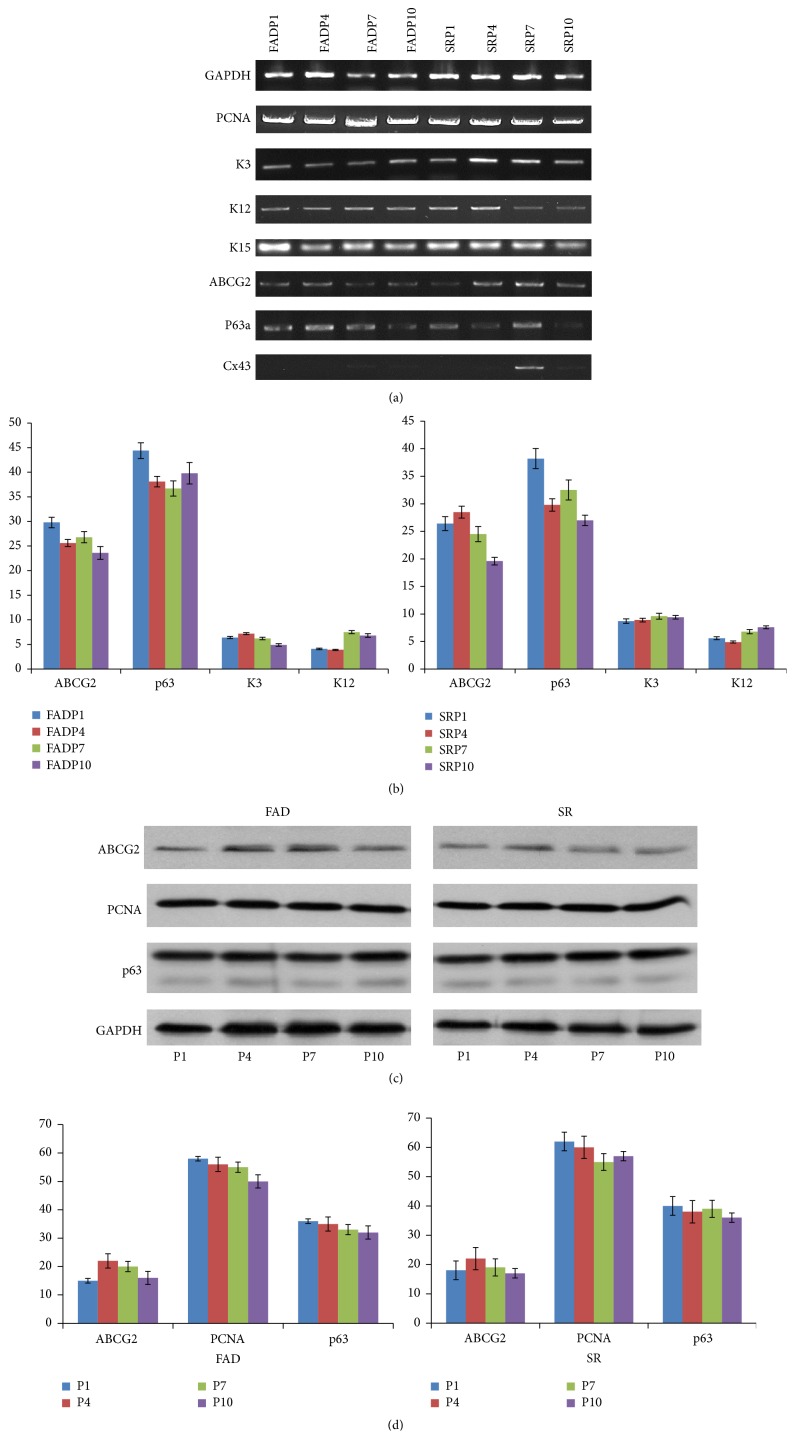
Gene expression and protein expression in human limbal epithelial cells. (a) Gene expression analysis of human limbal epithelial cells. PCR data shows that the cells cultured in both FAD medium and SR medium show high level expression of proliferation transcript PCNA in P1, P4, P7, and P10. The cells in both media show positive expression of cytokeratin 3 and cytokeratin 12, which implies their limbus origin. The positive expression of basal layer epithelial cell marker, cytokeratin 15, was also observed in both cultures. ABCG2 and ΔNp63*α* expression was detected in both cultures; their expression was observed to be decreased slightly after passage 7 (P7) in both culture media. The low level of connexin 43 (Cx43) expression was observed in both cultures and implied low level of epithelial cell differentiation in both media. (b) Quantitative real-time PCR analysis of human limbal epithelial cells cultured in SR and FAD media. Real-time PCR was performed on epithelial cells cultured using both FAD and SR media. Housekeeping gene GAPDH was used as an internal control. Analysis of the limbal epithelial stem cell markers p63 and ABCG2 mRNA found that there was no significant difference (*p* > 0.05, *n* = 5) in expression between epithelial cells grown in FAD and SR media. Real-time analysis of the corneal epithelial differentiation marker cytokeratin 3 and connexin 43 was performed. All these markers were barely detectable in both FAD and SR cell cultures, and, surprisingly, there is no significant difference (*p* > 0.05, *n* = 5) of expression level between these two cultures. All the real-time PCR experiments were carried out in triplicate. (c) Western blot assay of human limbal epithelial cells cultured in FAD and SR media. The expression of proliferation marker (PCNA) and potential epithelial stem cells markers (p63 and ABCG2) was evaluated using western blot. Using monoclonal antibody clone 4A4, the p63 protein (70 KD) was detected at high expression level in SR medium. The expression of ABCG2 was detected in each passage of cells cultured in SR medium. PCNA, the cell nucleus antigen, a cell proliferation marker, was also detected in epithelial cells cultured in SR medium. (d) The expression of ABCG2, p63, and PCNA was quantified and plotted using semiquantitative intensity measurement. The blot density of ABCG2, p63, and PCNA was measured and normalized to the level of GAPDH.

**Figure 4:**
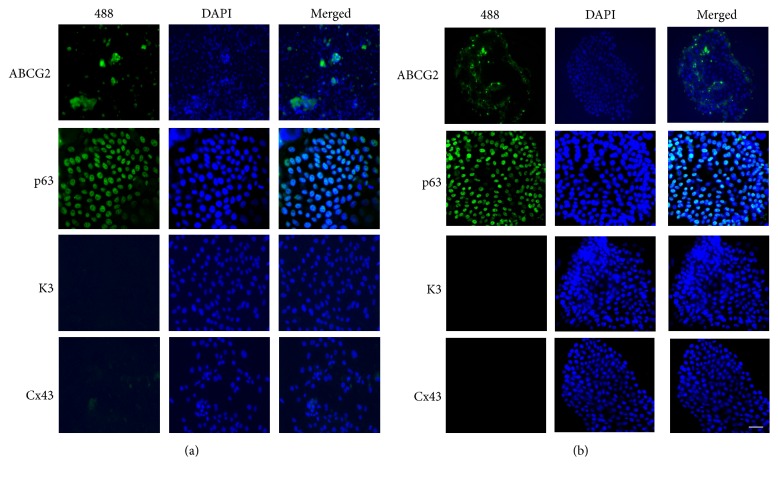
Expression of epithelial stem cell makers and differentiation markers in human limbal epithelial cells. The human limbal epithelial cells were strongly stained with ABCG2 and p63 (green) in SR and FAD media. The cells maintained in SR and FAD media showed weak staining for connexin 43 (Cx43) and cytokeratin 3 (K3). (a) SR medium and (b) FAD medium. Original magnification: (a) ×10; (b) ×10 (scale bars: 100 *μ*m). The studies were carried out in triplicate, and representative figures are shown here.

To double confirm the quantitative gene expression and immunofluorescent staining results, the expression of potential epithelial stem cells markers (p63 and ABCG2) and proliferation marker (PCNA) was evaluated using western blot. Using monoclonal p63 antibody (clone 4A4, Santa Cruz), the p63 protein (70 KD, ΔN*α* isoform) was detected at high expression level in SR medium. The expression of ABCG2 (70 KD) was detected in each passage of cells cultured in SR medium. PCNA, proliferation marker, was also detected in cells cultured in SR medium (Figure 3(c)). And the expression levels of p63, ABCG2, and PCNA were similar in 10 passages with semiquantitative intensity measurement (Figure 3(d)) using GAPDH as internal control.

## 4. Discussion

Epithelial cells cultured on 3T3 feeder layer have been successfully derived and used for treatment of various clinical situations, such as severe burns, diabetic foot ulcers, skin defects, and oral mucosa defects [13, 14, 21, 22]. The first transplantation of limbal epithelial stem cells was described in 1997 by Pellegrini et al. [10]. During the past decades, several different culture methods have been developed, including culture of limbal epithelial cells on human amniotic membrane (HAM) with or without 3T3 feeder layer [23–26], culture of epithelial cells on temperature-responsive plates [27], and culture of epithelial cells with commercial serum-free culture medium. As recent report indicates [28] that the clinical outcome of cornea/limbus epithelial cells transplantation depends on whether the limbal stem cells are preserved in the cell culture, it was pointed out that holoclones were only retained in FAD + 3T3 culture system [4, 11–16, 29, 30]. As this culture protocol involves using FBS, there is growing safety concerns that FBS is poorly defined animal products and has a potential of transmitting animal-derived diseases to patients [31]. Therefore there is increasing need to develop animal product-free and FBS-free culture medium to replace traditional FAD medium [32, 33].

In this study, we developed a novel serum-free culture medium, in which KnockOut SR replaced FBS. The phenotypes of limbal epithelial stem cells in this novel serum-free culture medium were evaluated by immunostaining with antibodies for proposed stem cell markers (p63, ABCG2) and differentiation markers (cytokeratin 3, connexin 43).

The nuclear transcript factor p63 was previously proposed to be a marker of epithelial stem cells, and ΔN*α* is the predominant isoform of p63 isoforms in these cells [34, 35]. Our results are consistent with these previous reports; nuclear p63 was strongly expressed in limbal cell culture, which was shown by immunostaining, real-time PCR, and western blot. The presence of p63 in limbal cell culture indicates their high proliferative and self-renewal potential. ABCG2, a member of the ATP binding cassette (ABC) transporters, originally known as breast cancer resistant protein 1 (BCRP1), has been proposed as another putative stem cell marker for adult stem cells, including limbus epithelial stem cells [36]. The high expression of ABCG2 in the SR medium was evidenced by immunostaining and real-time PCR.

K3 and K12 are well known as corneal specific markers [1, 37]. Consistently, our immunostaining and real-time PCR results showed that the cells were K3 and K12 negative, confirming their limbus origin. Connexin 43 is a member of the gap junction proteins family; it allows direct diffusion of low molecular weight solutes between neighboring cells. Connexin 43 was reported to be expressed by differentiated epithelial cells [38–40], and the absence of these intercellular communication molecules may be a feature of epithelial stem cells.

In our results, a greater percentage of cells expressed p63 and ABCG2, while few cells express differentiation markers K3/K12 and CX43. Thus human limbal epithelial stem cells in SR serum-free medium displayed similar phenotypes and maintain undifferentiated conditions, compared to FBS medium.

In conclusion, using KnockOut SR replacing FBS, our novel serum-free medium maintains human corneal epithelial cells growth and proliferation and retains epithelial stem cells in their undifferentiated phenotype. This novel serum-free culture method is supplement-defined and relatively easy to control. It has great potential to be used in clinic limbal epithelial cell transplantation and tissue regeneration.

## References

[1] Schermer A., Galvin S., Sun T.-T. (1986). Differentiation-related expression of a major 64K corneal keratin in vivo and in culture suggests limbal location of corneal epithelial stem cells. *Journal of Cell Biology*.

[2] Cotsarelis G., Cheng S.-Z., Dong G., Sun T.-T., Lavker R. M. (1989). Existence of slow-cycling limbal epithelial basal cells that can be preferentially stimulated to proliferate: implications on epithelial stem cells. *Cell*.

[3] Huang A. J. W., Tseng S. C. G. (1991). Corneal epithelial wound healing in the absence of limbal epithelium. *Investigative Ophthalmology and Visual Science*.

[4] Pellegrini G., Golisano O., Paterna P. (1999). Location and clonal analysis of stem cells and their differentiated progeny in the human ocular surface. *Journal of Cell Biology*.

[5] Thoft R. A., Friend J. (1983). The X, Y, Z hypothesis of corneal epithelial maintenance. *Investigative Ophthalmology and Visual Science*.

[6] Buck R. C. (1985). Measurement of centripetal migration of normal corneal epithelial cells in the mouse. *Investigative Ophthalmology and Visual Science*.

[7] Henderson T. R. M., Coster D. J., Williams K. A. (2001). The long term outcome of limbal allografts: the search for surviving cells. *British Journal of Ophthalmology*.

[8] Daya S. M., Watson A., Sharpe J. R. (2005). Outcomes and DNA analysis of ex vivo expanded stem cell allograft for ocular surface reconstruction. *Ophthalmology*.

[9] Rheinwald J. G., Green H. (1975). Serial cultivation of strains of human epidermal keratinocytes: the formation of keratinizing colonies from single cells. *Cell*.

[10] Pellegrini G., Traverso C. E., Franzi A. T., Zingirian M., Cancedda R., De Luca M. (1997). Long-term restoration of damaged corneal surfaces with autologous cultivated corneal epithelium. *The Lancet*.

[11] Barrandon Y., Green H. (1987). Three clonal types of keratinocyte with different capacities for multiplication. *Proceedings of the National Academy of Sciences of the United States of America*.

[12] Rochat A., Kobayashi K., Barrandon Y. (1994). Location of stem cells of human hair follicles by clonal analysis. *Cell*.

[13] Pellegrini G., Ranno R., Stracuzzi G. (1999). The control of epidermal stem cells (holoclones) in the treatment of massive full-thickness burns with autologous keratinocytes cultured on fibrin. *Transplantation*.

[14] Ronfard V., Rives J.-M., Neveux Y., Carsin H., Barrandon Y. (2000). Long-term regeneration of human epidermis on third degree burns transplanted with autologous cultured epithelium grown on a fibrin matrix. *Transplantation*.

[15] Barbaro V., Testa A., Di Iorio E., Mavilio F., Pellegrini G., De Luca M. (2007). C/EBP*δ* regulates cell cycle and self-renewal of human limbal stem cells. *Journal of Cell Biology*.

[16] Dellambra E., Golisano O., Bondanza S. (2000). Downregulation of 14-3-3*σ* prevents clonal evolution and leads to immortalization of primary human keratinocytes. *Journal of Cell Biology*.

[17] Zheng X., Baker H., Hancock W. S., Fawaz F., McCaman M., Pungor E. (2006). Proteomic analysis for the assessment of different lots of fetal bovine serum as a raw material for cell culture. Part IV. Application of proteomics to the manufacture of biological drugs. *Biotechnology Progress*.

[18] Tuschong L., Soenen S. L., Blaese R. M., Candotti F., Muul L. M. (2002). Immune response to fetal calf serum by two adenosine deaminase-deficient patients after T cell gene therapy. *Human Gene Therapy*.

[19] De Paiva C. S., Pflugfelder S. C., Li D.-Q. (2006). Cell size correlates with phenotype and proliferative capacity in human corneal epithelial cells. *Stem Cells*.

[20] Chen Z., De Paiva C. S., Luo L., Kretzer F. L., Pflugfelder S. C., Li D.-Q. (2004). Characterization of putative stem cell phenotype in human limbal epithelia. *Stem Cells*.

[21] Gallico G. G., O'Connor N. E., Compton C. C. (1984). Permanent coverage of large burn wounds with autologous cultured human epithelium. *The New England Journal of Medicine*.

[22] Romagnoli G., De Luca M., Faranda F. (1990). Treatment of posterior hypospadias by the autologous graft of cultured urethral epithelium. *The New England Journal of Medicine*.

[23] Tsai R. J.-F., Li L.-M., Chen J.-K. (2000). Reconstruction of damaged corneas by transplantation of autologous limbal epithelial cells. *The New England Journal of Medicine*.

[24] Schwab I. R. (1999). Cultured corneal epithelia for ocular surface disease. *Transactions of the American Ophthalmological Society*.

[25] Koizumi N., Inatomi T., Suzuki T., Sotozono C., Kinoshita S. (2001). Cultivated corneal epithelial transplantation for ocular surface reconstruction in acute phase of Stevens-Johnson syndrome. *Archives of Ophthalmology*.

[26] Grueterich M., Espana E., Tseng S. C. G. (2002). Connexin 43 expression and proliferation of human limbal epithelium on intact and denuded amniotic membrane. *Investigative Ophthalmology and Visual Science*.

[27] Nishida K., Yamato M., Hayashida Y. (2004). Functional bioengineered corneal epithelial sheet grafts from corneal stem cells expanded ex vivo on a temperature-responsive cell culture surface. *Transplantation*.

[28] Rama P., Matuska S., Paganoni G., Spinelli A., De Luca M., Pellegrini G. (2010). Limbal stem-cell therapy and long-term corneal regeneration. *New England Journal of Medicine*.

[29] Barrandon Y., Green H. (1987). Cell migration is essential for sustained growth of keratinocyte colonies: the roles of transforming growth factor-*α* and epidermal growth factor. *Cell*.

[30] Pellegrini G., Rama P., Mavilio F., De Luca M. (2009). Epithelial stem cells in corneal regeneration and epidermal gene therapy. *The Journal of Pathology*.

[31] Halme D. G., Kessler D. A. (2006). FDA regulation of stem-cell-based therapies. *New England Journal of Medicine*.

[32] Yokoo S., Yamagami S., Usui T., Amano S., Araie M. (2008). Human corneal epithelial equivalents for ocular surface reconstruction in a complete serum-free culture system without unknown factors. *Investigative Ophthalmology and Visual Science*.

[33] Lekhanont K., Choubtum L., Chuck R. S., Sa-Ngiampornpanit T., Chuckpaiwong V., Vongthongsri A. (2009). A serum- and feeder-free technique of culturing human corneal epithelial stem cells on amniotic membrane. *Molecular Vision*.

[34] Pellegrini G., Dellambra E., Golisano O. (2001). p63 identifies keratinocyte stem cells. *Proceedings of the National Academy of Sciences of the United States of America*.

[35] Iorio E. D., Barbaro V., Ruzza A., Ponzin D., Pellegrini G., De Luca M. (2005). Isoforms of ΔNp63 and the migration of ocular limbal cells in human corneal regeneration. *Proceedings of the National Academy of Sciences of the United States of America*.

[36] Schlötzer-Schrehardt U., Kruse F. E. (2005). Identification and characterization of limbal stem cells. *Experimental Eye Research*.

[37] Liu C.-Y., Zhu G., Converse R. (1994). Characterization and chromosomal localization of the cornea-specific murine keratin gene Krt1.12. *The Journal of Biological Chemistry*.

[38] Matic M., Petrov I. N., Chen S., Wang C., Dimitrijevich S. D., Wolosin J. M. (1997). Stem cells of the corneal epithelium lack connexins and metabolite transfer capacity. *Differentiation*.

[39] Dong Y., Roos M., Gruijters M. (1994). Differential expression of two gap junction proteins in corneal epithelium. *European Journal of Cell Biology*.

[40] Wolosin J. M., Xiong X., Schütte M., Stegman Z., Tieng A. (2000). Stem cells and differentiation stages in the limbo-corneal epithelium. *Progress in Retinal and Eye Research*.

